# Nanocarrier-Based Approaches for the Efficient Delivery of Anti-Tubercular Drugs and Vaccines for Management of Tuberculosis

**DOI:** 10.3389/fphar.2021.749945

**Published:** 2021-12-21

**Authors:** Amarjitsing Rajput, Satish Mandlik, Varsha Pokharkar

**Affiliations:** Department of Pharmaceutics, Poona College of Pharmacy, Bharti Vidyapeeth Deemed University, Pune, India

**Keywords:** tuberculosis, *Mycobacterium tuberculosis*, drug delivery systems, nanocarriers, vaccines

## Abstract

Drug-resistant species of tuberculosis (TB), which spread faster than traditiona TB, is a severely infectious disease. The conventional drug therapy used in the management of tuberculosis has several challenges linked with adverse effects. Hence, nanotherapeutics served as an emerging technique to overcome problems associated with current treatment. Nanotherapeutics helps to overcome toxicity and poor solubility issues of several drugs used in the management of tuberculosis. Due to their diameter and surface chemistry, nanocarriers encapsulated with antimicrobial drugs are readily taken up by macrophages. Macrophages play a crucial role as they serve as target sites for active and passive targeting for nanocarriers. The surface of the nanocarriers is coated with ligand-specific receptors, which further enhances drug concentration locally and indicates the therapeutic potential of nanocarriers. This review highlights tuberculosis’s current facts, figures, challenges associated with conventional treatment, different nanocarrier-based systems, and its application in vaccine development.

## Introduction

### Tuberculosis as an Infectious Disease

Tuberculosis (TB) is a disease caused by the bacteria *Mycobacterium tuberculosis* (Mtb), which is passed from person to person through droplets generated by infected people (Sinha et al., 2016; WHO, 2020)*.*

### Current Facts and Figures as per the World Health Organization

According to the World Health Organization’s (WHO) annual 2020 global TB report, an estimated 10 million people were infected with tuberculosis in 2019, with 1.2 million fatalities, making it one of the world’s most infectious diseases (WHO, 2020).

### Challenges in Tuberculosis Diagnosis and Treatment

The current treatments are restricted because of low effectiveness, more toxicity (hepatotoxicity), extended duration, patient non-compliance, development of multi-drug resistance (MDR), and extensively drug resistance (XDR) tuberculosis. MDR is the resistance of Mtb strains to at least rifampicin and isoniazid, which are considered cornerstone compounds for managing TB. XDR is TB that occurs due to Mtb strains that fulfil the definition of MDR/RR (Rifampicin resistance)-TB (WHO, 2021).

Hence, targeting either the host or pathogen decreases the treatment duration and limits the appearance of antibiotic resistance in Mtb (Li and Nikaido, 2004; Khan et al., 2017). Additionally, the development of novel drug delivery methods could serve as promising systems in treating MDR/XDR tuberculosis (Dua et al., 2018). The challenges associated with tuberculosis treatment and current facts and figures are as shown in Supplementary Figure S1.

## Nanocarrier-Based Systems for Treatment of Tuberculosis

**FIGURE 1:**
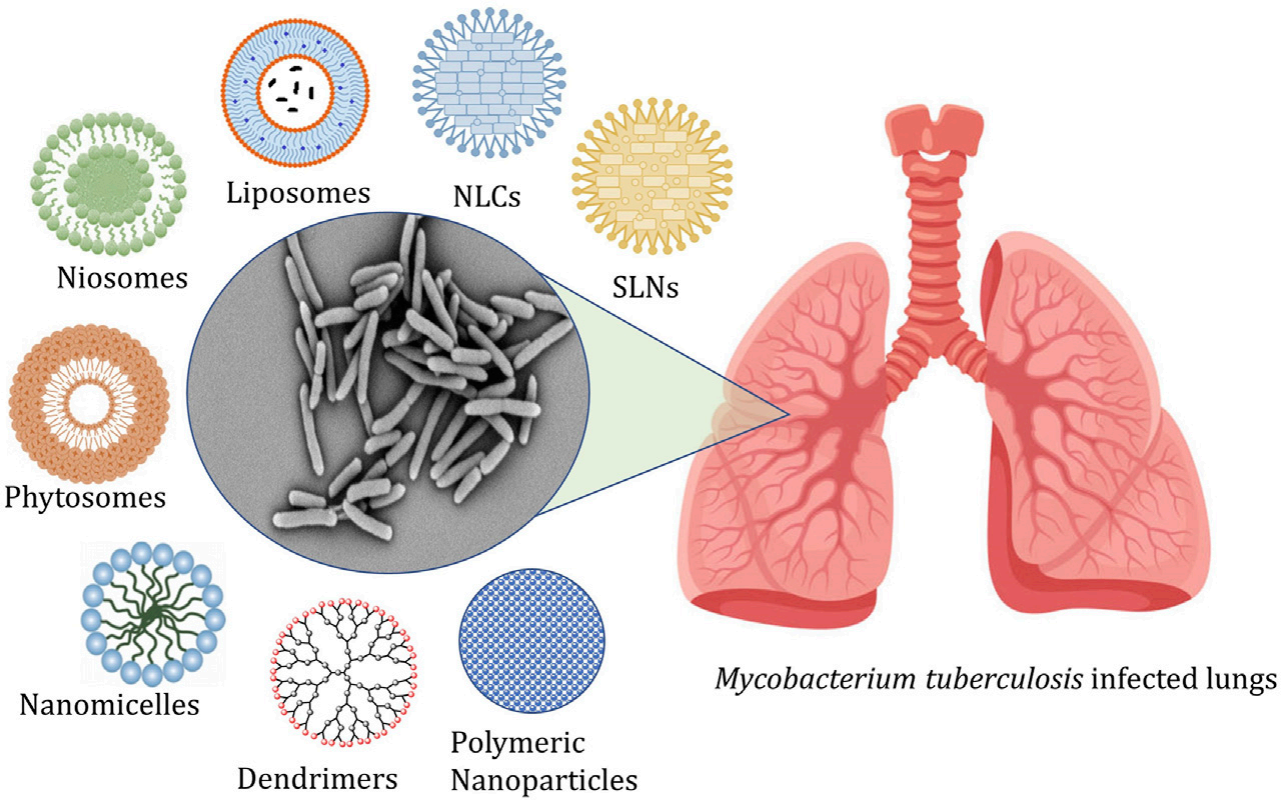
Represents the targeting of alveolar macrophages and granulomas using nanocarriers administered by different routes.

The conventional treatment of tuberculosis consists of administering first, second, and third-line drugs for a prolonged period. The efficient treatment needs a minimum of a 6 months course comprising a combination of antibiotics along with at least three bactericidal compounds (Patil, 2016). This is a leading contributory parameter in the relapses of the infection and the advent of MDR-TB and XDR-TB (Chan and Iseman, 2008). The marketed products available for conventional therapy are linked with numerous challenges like poor penetrability, metabolic instability, poor solubility, and associated adverse effects (Shivangi and Meena, 2018; Raza et al., 2019). Thus, these problems of conventional therapy resulted in a paradigm shift to nano carrier-based drug delivery systems (Patil et al., 2015; Costa et al., 2018). The different types of nanocarriers explored for management of tuberculosis are shown in Figure 1.

### Role of Nanocarriers

Nanomedicine is considered the most recent invention in biomedical science, impacting diagnosis, management, and deterrence of several infectious diseases, including tuberculosis. Nanomedicine aids in the delivery of hydrophilic and hydrophobic compounds, macromolecules (intracellularly), and site-specific delivery of actives to cells or tissues (McAllister et al., 2007).

### Advantages of Nanocarriers

Nanocarriers provide several benefits like significant decrease in size, high surface volume ratio, site-specificity, and being functionalized to alter the therapeutic potential of drugs (Costa-Gouveia et al., 2017; Patil et al., 2018). Moreover, the specific nanocarriers own intrinsic antimicrobial properties and act as a vehicle for different anti-tubercular substances, thus aiding in decreasing dosing regimen and related adverse effects (Costa-Gouveia et al., 2017). Various researchers have explored several nanocarriers for tuberculosis management and are exemplified below.

### Lipid-Based Systems

#### Solid Lipid Nanoparticles

SLN is a nano-diameter lipid particulate system that has attracted more focus due to its specific distribution, biocompatibility, enhanced encapsulation of drugs, controlled delivery, and increment in the bioavailability of entrapped compounds (Pandey et al., 2005).

Singh et al. developed streptomycin sulfate-laden SLN for enhancement of oral bioavailability. Streptomycin sulfate (STRS)-laden SLN formulated using cold homogenization technique showed particle diameter (218.1 ± 15.46 nm), drug loading (30%), encapsulation efficiency (51.17 ± 0.95%), and mucus penetration characteristics. Compared to pure STRS, STR-SLNs showed a 3-fold decrease in the minimum inhibitory concentration (MIC) against Mtb H37RV. It offered better action against the other two species, namely *M. bovis* BCG and Mtb H37RV, than pure STRS. The acute cytotoxicity studies indicated the safety of developed formulation both *in vitro* and *in vivo.* Finally, a pharmacokinetics study of developed formulation in rats showed a significant increase (160–710%) in oral absorption and bioavailability compared to pure STRS (Singh et al., 2021).

#### Nanostructured Lipid Carrier

Nanostructured lipid carrier (NLC) is the next generation of lipid carrier systems mainly designed to surpass the shortcomings associated with SLN. NLC combines solid and liquid lipids, thus reducing crystallinity and a loosely arranged matrix system (Garg et al., 2016a; Pinheiro et al., 2016).

Patil et al. developed a clofazimine-loaded NLC to improve its bioavailability and safety in TB (Patil and Deshpande, 2021). *In vitro* hemocompatibility tests, cell viability tests on macrophage J774 cell lines, and *in vivo* acute inhalation toxicity analysis were used to investigate the safety profile of clofazimine-NLC and mannosylated-clofazimine-NLC. Acute toxicity data collected *in vivo* for 14 days showed no physiological or behavioral impairments and no mortality. The pharmacokinetic results indicated the highest amount of clofazimine (C_max_) of 35.44 ± 0.34 μg/g from mannosylated-clofazimine NLC posts 48 h. Mannosylated-clofazimine NLC exhibited a maximum (AUC_0–∞_) of 2,691.83 h μg/ml in the lungs, suggesting two times more bioavailability than clofazimine dispersion. As a result, mannosylated NLC can be a safe and effective system for delivery clofazimine in TB. Supplementary Figure S2 showed lipidic nanocarriers used in Mtb targeting.

#### Lipid-Drug Conjugate

Lipid drug conjugate (LDC) are lipidic prodrugs (Lambert, 2000) that consist of drugs bound to the lipidic moiety like fatty acid, phosphoglyceride, or a diglyceride bound together covalently or non-covalently (Adhikari et al., 2017).

Pandit et al. developed and studied intracellular trafficking of lipid-drug conjugate nanoparticles containing isoniazid (Pandit et al., 2020). LDC containing isoniazid was formulated using cold homogenization technique and characterized for various parameters. The developed LDC had a drug load of 92.73 ± 6.31% w/w and a monodisperse particle size of 124.60 ± 5.56 nm. Furthermore, the LDC revealed the ability to promote intracellular trafficking into endosomal and lysosomal vesicles, showing that CD63, LAMP-2, EEA1, and Rab 11 are involved in Mtb pathogenesis. Thus, prepared LDC showed the capability to enhance the intracellular transport of a highly water-soluble molecule like isoniazid (Pandit et al., 2020).

#### Lipid-polymer Hybrid Nanoparticles

Lipid polymer hybrid nanoparticle (LPHNPs) acts as potential drug delivery system that uses the drug delivery action of liposomes and polymeric nanoparticles to overcome their shortcomings (Garg et al., 2017; Garg et al., 2018; Jadon et al., 2021).

Bhardwaj et al. developed LPHNPs loaded with isoniazid using the spray drying technique. The formulated isoniazid LPHNPs were spherical with 111.81 ± 1.2 nm particle size and 0.189 ± 1.4 polydispersity index (PDI), suggesting their uptake by alveolar macrophages. The developed formulation was proven to have more uptake potential by J774A.1 cells than free isoniazid. Finally, an *in vivo* study in mice using dry powder insufflator containing isoniazid was more clearly detected in the plasma post 24 h administration of isoniazid loaded LPHNPs than free isoniazid (not traced post 24 h). Thus, isoniazid-loaded LPHNPs serve as a suitable carrier for pulmonary delivery in the management of tuberculosis (Bhardwaj et al., 2016).

### Emulsion-Based Systems

#### Microemulsion

Microemulsions are transparent and isotropic drug delivery systems that are thermodynamically stable. They consist of a surfactant and co-surfactant that alter at least two mutually incompatible solvents, classically water and oil (Talmon and Prager, 1977; You et al., 2017).

Kaur et al. (2015) developed Brig 96 microemulsion containing both hydrophobic and hydrophilic drugs for synergistic action. The microemulsion was prepared to entrap anti-tubercular compounds to overcome the stability problems of rifampicin (RIF) in the presence of isoniazid (INH) and pyrazinamide (PZA). According to the release kinetics investigation, INH and PZA followed a diffusional (Fickian) while RIF followed an anomalous release mechanism. The antimicrobial study showed that compounds when used in combination provided potential antimicrobial activity. Thus, the study suggested that microemulsions serve as an excellent carrier in multiple drug therapy.

#### Nanoemulsion

Nanoemulsion is a potential carrier for enhancing the bioavailability of anti-tubercular compounds mainly *via* the oral route. The nanoemulsion laden with anti-tubercular molecules can freely transport across the biological barriers and therefore decrease the load of Mtb (Patil et al., 2018).

Henostroza et al. developed rifampicin-loaded cationic nanoemulsion with specific surface alteration using chitosan and polymyxin B. Rifampicin nanoemulsion containing chitosan and rifampicin nanoemulsion containing polymyxin B have both shown particle size around 150 nm and zeta potential values of +51.3 mV and +5.5 mV, respectively. *In vitro* mucoadhesion study results reported an electrostatic interaction among the cationic nanoemulsion and negatively charged mucin. This results in enhancing the effectiveness of the developed preparation by improving its residence time at the target site for treating ocular tuberculosis (Bazán Henostroza et al., 2020).

### Vesicular Drug Delivery Systems

#### Liposomes

Liposomes are biocompatible, spherical, lipid bilayers vesicles assembled with an inner core comprising an aqueous phase. The reported liposomal carriers have shown effectiveness against multi-drug and extensively drug-resistant strains of *Mycobacterium* (Bekraki, 2020)*.* Liposomes have also been a helpful system for vaccine delivery in managing tuberculosis (Tyagi et al., 2015; Tyagi et al., 2016; Diogo et al., 2019; Tandel et al., 2020; Chaudhari et al., 2021).

Tian et al. formulated and evaluated the pCMFO antigen-loaded dimethyl-dioctadecyl-ammonium (DDA) liposome with and without mono-phosphoryl lipid A (MPLA) and trehalose 6,6′-dibehenate (TDB). The *in vivo* studies on experimental mice were performed with different treatments: pCMFO, pCMFO/DDA, or pCMFO/DMT. The immunogenicity and protective efficiency of BCG-vaccinated mice against Mtb infection were also compared and analyzed. The dimethyl-dioctadecyl-ammonium liposome alone, or the addition of TDB or MPLA to the DDA liposome, elicited early interleukin-17 and interferon responses in vaccinated mice. Thus, the controlled release function of the DMT liposome may be connected to the enhanced efficacy of DMT (adjuvant) vaccination against TB (Tian et al., 2018).

#### Niosomes

Niosomes exhibit pharmaceutically acceptable properties such as non-toxicity, stability, sustained action, biodegradability, modification of drug distribution, and potential for improved drug bioavailability (Khan and Irchhaiya, 2016; Khoee and Yaghoobian, 2017).

Ethionamide and D-Cycloserine dual drug-loaded self-assembled niosomes have been produced by Kulkarni et al. for the successful treatment of multidrug-resistant tuberculosis. The niosomes were optimized by Box Behnken design and characterized for atomic force microscopy, *in vitro* hemodialysis, osmotic shock, and antimicrobial studies. The hemodialysis studies proved to offer safe intravenous administration of dual drug-loaded niosomes. The MIC of dual drug-loaded niosomes was the lowest *vis-a-vis* pure drug and single drug-loaded niosomes. This effectiveness was observed due to the slow release of lipophilic ethambutol and the initial burst release of D-Cycloserine. Thus, the synergetic effect of a dual drug embedded in niosomes was an effective treatment for tuberculosis (Kulkarni et al., 2019).

Figure 1 represents the targeting of alveolar macrophages and granulomas using nanocarriers administered by different routes, and Table 1 shows a list of nanocarriers for tuberculosis management.

**TABLE 1:** List of nanacarriers useful in tuberculosis management.

Type of nanocarrier	Antitubercular drug used	References
Liposomes	Rifampicin	Vyas et al. (2004), Changsan et al. (2009), Zaru et al. (2009), Manca et al. (2012), Patil (2016)
	Rifapentine proliposomes	Patil-Gadhe and Pokharkar (2014)
	Isoniazid	Nkanga et al. (2017)
	Pyrazinamide	El-Ridy et al. (2007), Rojanarat et al. (2012)
	Ethambutol	Wiens et al. (2004)
	Streptomycin	Vladimirsky and Ladigina (1982), Su et al. (2018)
Niosomes	Rifampicin	Jain et al. (2006), Rani et al. (2010), Khan et al. (2020)
	Isoniazid	Singh et al. (2011)
	Ethionamide	Kulkarni et al. (2019)
	Gatifloxacin	Rani et al. (2010)
	Ciprofloxacin	Moazeni et al. (2010)
Solid lipid nanoparticles (SLN)	Rifampicin	Vieira et al. (2018)
	Isoniazid	Ma et al. (2021)
	Rifampicin loaded mannosylated SLN	Truzzi et al. (2020)
	Bedaquiline	De Matteis et al. (2018)
Nanostructured lipid carriers (NLC)	Rifabutin	Pinheiro et al. (2016)
	Rifampicin	Carneiro et al. (2019)
	Manosylated cationic NLC of rifampicin	Song et al. (2015)
	Linezolid	Makled et al. (2021)
Nanomicelles	Rifampicin loaded chitosan nanomicelles	Praphakar et al. (2017)
	Isoniazid and Rifampicin	Praphakar et al. (2018)
Chitosan nanoparticles	Bedaquiline	Rawal et al. (2018)
	Levofloxacin	Shah et al. (2021)
	Rifampicin	Rawal et al. (2017)
	Isoniazid and Rifampicin	Garg et al. (2016b)
Dendrimers	Rifampicin	Bellini et al. (2015)
	Isoniazid	Rodrigues and Shende, (2020)
Carbon nanotubes	Isoniazid	Zomorodbakhsh et al. (2020)
	Pyrazinamide	Mani et al. (2015)
Dual loaded nanoparticles	Rifampicin and Curcumin	Jahagirdar et al. (2020)

#### Phytosomes

Phytosomes are commonly used to enhance the bioavailability of herbal extracts (Sharma and Roy, 2010; Awasthi et al., 2011). The anti-tubercular herbal extract of *Glycyrrhiza glabra* was embedded into the inhalable liposomes and showed significant inhibition of mycobacteria in tuberculosis-induced mice (Viswanathan et al., 2019).

#### Transferosomes

Transferosomes are ultra-deformable elastic vesicles composed of phospholipids and an edge activator (Rajan et al., 2011; Fernández-García et al., 2020).

Van *et al.* formulated the lipidic vesicles of artemisone, clofazimine, and decoquinate for the topical treatment of cutaneous tuberculosis. The vesicles comprising 1% each of active pharmaceutical ingredients were formulated by thin-film hydration technique. The various studies like hot stage microscopy and differential scanning isothermal calorimeter have shown minimal incompatibility among the APIs and other components of vesicles. Finally, developed formulation effectiveness was studied against the Mtb H37Rv laboratory strain and showed highest percentage inhibition with niosomes containing 1% clofazimine (van Zyl and Viljoen, 2019).

### Other Nanocarriers

#### Dendrimers

Dendrimers are nanosized and symmetrically ordered branched polymers with several functional groups significantly used for targeted drug delivery. Due to their unique structure, dendrimers are suitable for encapsulating anti-tubercular drugs (Ghavami and Sardari, 2011).

Ahmed et al. developed and explored the effectiveness of a surface-modified 4.0 G PAMAM dendrimer as an innovative drug delivery system loaded with rifampicin to manage tuberculosis. The rifampicin was encapsulated into dendrimers using a simple dissolution solvent evaporation technique and evaluated for different parameters. The percentage coverage of 4.0 G PAMAM dendrimer peripheral with PEG was attained between 38–100%. The entrapment efficiency of native dendrimer was found to be 7.5% (W/W), and PEGylated dendrimer was >60% (w/w). The PEGylated dendrimers indicated a slow-release rate compared to the native formulation and free drug. The PEGylation of a dendrimer showed negligible toxicity for formulation with 100% functionalization. Thus, formulated PEGylated 4.0 G PAMAM dendrimers are proven to be the choice of drug carrier with high loading, negligible toxicity, and extended-release pattern for rifampicin (Ahmed et al., 2021).

An isoniazid embedded dendrimer complexation with copper nanoclusters showed synergism in dose reduction on the *Mycobacterium tuberculosis* H37Ra strain. This nanocluster could offer a novel alternative in treating tuberculosis (Rodrigues and Shende, 2020). Bellini et al. implemented molecular dynamics simulations to investigate rifampicin conjugation with a G4-PAMAM, a fourth-generation poly-amido-amine dendrimer for effective drug delivery, emphasizing physiological stability and pH-dependent release (Bellini et al., 2015).

#### Carbon Nanotubes

Carbon nanotubes (CNTs) are hollow tubular/cylindrical nanosized carbon atom structures that are hexagonally organized (Popov, 2004; Grumezescu, 2016). Many studies explored the possibility of using carbon nanotubes as effective carriers for delivering anti-tubercular compounds and overcoming tuberculosis’s antibacterial resistance (Praphakar et al., 2018).

Chen et al. (2019) developed and characterized isoniazid-chitosan-carbon nanotubes. In the *in vitro* studies, optimized drug-loaded nanotubes effectively inhibited *Mycobacterium tuberculosis* colonies. The isoniazid-chitosan-carbon nanotubes significantly healed tuberculosis ulcers which were induced in experimental guinea pigs. The immunohistochemistry showed the lowest number of CD4+T cells concerning pure isoniazid. The constructed isoniazid chitosan CNTs prolonged the release of isoniazid and reduced cytotoxicity and inflammation.

#### Nanomicelles

Nanomicelles are versatile nanocarriers constructed from amphiphilic monomers. The polymeric nanomicelles can encapsulate both hydrophilic and lipophilic drugs, thus providing a platform for solubility enhancement of poorly soluble drugs (Cholkar et al., 2012; Trinh et al., 2017).

Praphakar et al. developed the rifampicin-loaded chitosan nanomicelles and characterized the *ex vivo* cellular uptake using U937 cells and internalization of the multi-drug micelles in the zebrafish model, which showed excellent results (Amarnath Praphakar et al., 2019). Fluorescence micrographs of nanomicelles showed significant entry into A549 cell lines and thus proved its therapeutic effectiveness for the management of tuberculosis (Praphakar et al., 2017).

### Application of Different Nanocarriers in Vaccine Development

Vaccines as a preventive measure have been formulated using killed or live attenuated microorganisms or components (like DNA or RNA) of a pathogen (Homma et al., 2013). Many vaccines are under development in different phases of clinical trials, namely AEC/BC02, MTBVAC, ID 93+GLA-SE, VPM 1002, RUTI, H56:IC31, Ad5Ag85A, M72 + AS01, DAR-901 booster, and TB-FLU-04L (Hussain et al., 2019). The BCG vaccine is made from a weakened strain of mycobacteria, and it uses the immune system to protect against the most severe forms of tuberculosis by stimulating T cell responses (Doherty and Andersen, 2005). VPM1002, a recombinant BCG (rBCG) vaccine, is one of the most advanced tuberculosis vaccines being tested in clinical trials.

The nanocarriers play a pivotal role in developing a vaccine that enhances the cellular and humoral immunological responses. The use of nanocarriers in vaccine development enhances antigen stability and immunogenicity and improves targeted delivery of vaccines. The nanocarriers, such as polymeric nanoparticles, liposomes, nanotubes, micelles, and virus-like particles, have been shown to be good vehicles for delivering antigens and acting as adjuvants (Hajizade et al., 2014; Mehdi et al., 2020).

The liposomes act as an immunological adjuvant and are extensively studied in vaccine development. The clinical trials have been conducted on many liposomal vaccines and received approval for effective tuberculosis management (Kim et al., 2014).

Mansury et al. formulated and evaluated the immunogenicity of the liposomes composed of the fusion protein (FP) of Mtb. It was observed that the optimized liposomal administration post-BCG vaccine contributed to the enhancement of γ interferon and interleukin-12, which confirms the enhancement of the BCG vaccine’s effectiveness (Mansury et al., 2019). Cationic liposomes can achieve the stability of subunit TB vaccines at the injection site for an extended period; it also provides solid electrostatic interactions with antigen-presenting sites and enhances immune responses (Khademi et al., 2018).

## Recent Developments and Future Prospects

The recent updates in the management of tuberculosis includes three drugs, namely bedaquiline (2012), delamanid (2014), and pretomanid (2019), with a novel mechanism of action that have received conditional regulatory approval for the treatment of MDR and XDR tuberculosis. WHO reported newer anti-tubercular drugs in clinical trial phases: contezoid, delpazolid, mcaozinone, pretomanid, sutezolid, OPC-167832, Q203, SQ109, TBA-7371, and TBI-166 (Hussain et al., 2019).

The Nix-TB is the first TB clinical trial to test a new drug combination; pretomanid, bedaquiline, and linezolid. It is also collectively known as the BPal regimen, predicated to cure XDR-TB in six to 9 months (Conradie et al., 2020). Multiple active targeting of nanocarriers to the macrophages using various ligands is one of the research strategies recently explored by different researchers (Baranyai et al., 2021). Further, advances in drug loading, characterization, and scalability are needed to develop optimized and scalable formulations for tuberculosis treatment. A multidisciplinary approach is required for effective and focused tuberculosis treatment (Doherty and Andersen, 2005).

## Conclusion

Tuberculosis is the most common cause of death around the world. More prevalence is due to the infectious process’ ability to grow and multiply within macrophages, leading to multi-drug resistance (MDR) and extensive drug resistance (XDR) tuberculosis. Nanotechnology offers novel avenues for tuberculosis diagnosis, treatment, and prevention, the most potential output being developing various nano carrier-based drug delivery systems and vaccines. Nanocarrier-based drug delivery systems improve bioavailability and biodistribution by reducing the dose and severity of accompanying adverse effects. Thus, to attain ground-breaking development and ensure the pulmonary administration of nanocarrier-based drug delivery systems for non-invasive clinical trials, multidisciplinary techniques are needed; nanotechnology, medicine, and engineering should work together.

## Abbreviations

CNTs, carbon nanotubes; DDA, dimethyl octadecyl-ammonium; DOT, direct observation therapy; FP, fusion protein; INH, isoniazid; LDC, lipid drug conjugate; LP, liposomes; MDR, multi-drug resistance; ME, microemulsion; MIC, minimum inhibitory concentration; Mtb, *Mycobacterium tuberculosis*; MWCNTs, multiwalled carbon nanotubes; NE, nanoemulsion; NLC, nanostructured lipid carrier; NMP, nano-microparticles; NPs, nanoparticles; PZA, pyrazinamide; RIF, rifampicin; RR, rifampicin resistance; SLM, solid lipid microparticles; STRS, streptomycin sulphate; SWCNTs, single-walled carbon nanotubes; TB, Tuberculosis; TDB, Trehalose 6,6-dibehenate; TEM, transmission electron microscopy; TPG, tocopheryl polyethylene glycol succinate; WHO, world health organization; XDR, extensive drug resistance; ZrO_2_, zirconia.
