# Supplementary figures and images for: Nanocarrier-Based Approaches for the Efficient Delivery of Anti-Tubercular Drugs and Vaccines for Management of Tuberculosis
Source: Front Pharmacol. 2021 Dec 21;12:749945. doi: 10.3389/fphar.2021.749945 (PMC8724553; doi:10.3389/fphar.2021.749945)

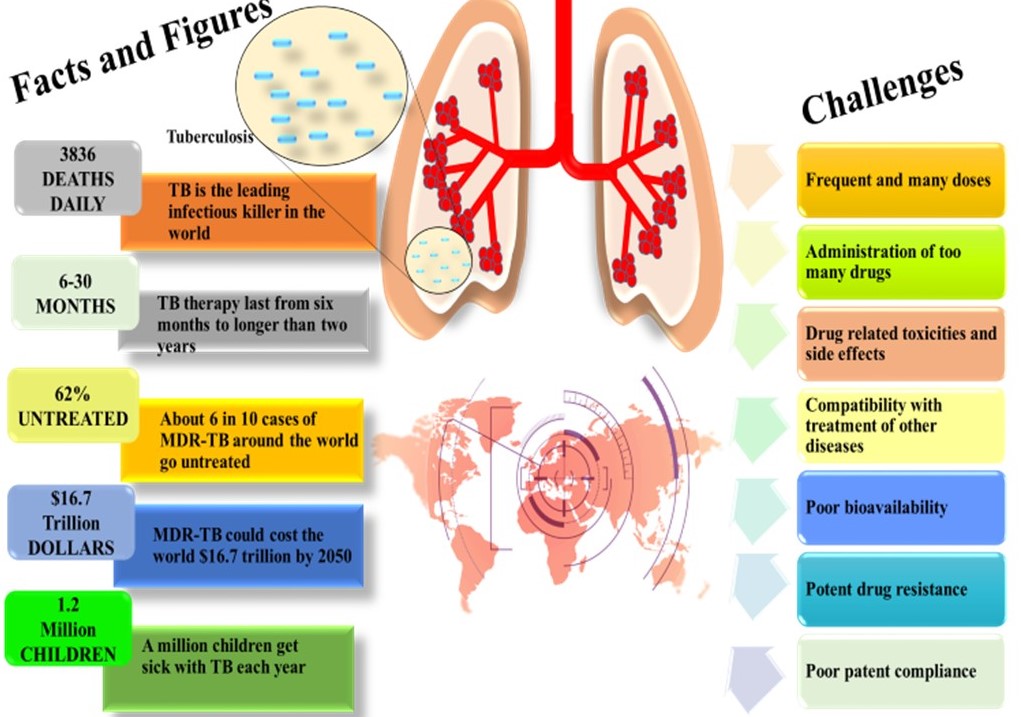

Supplement: Supplementary file 1 [file Image1.JPEG]

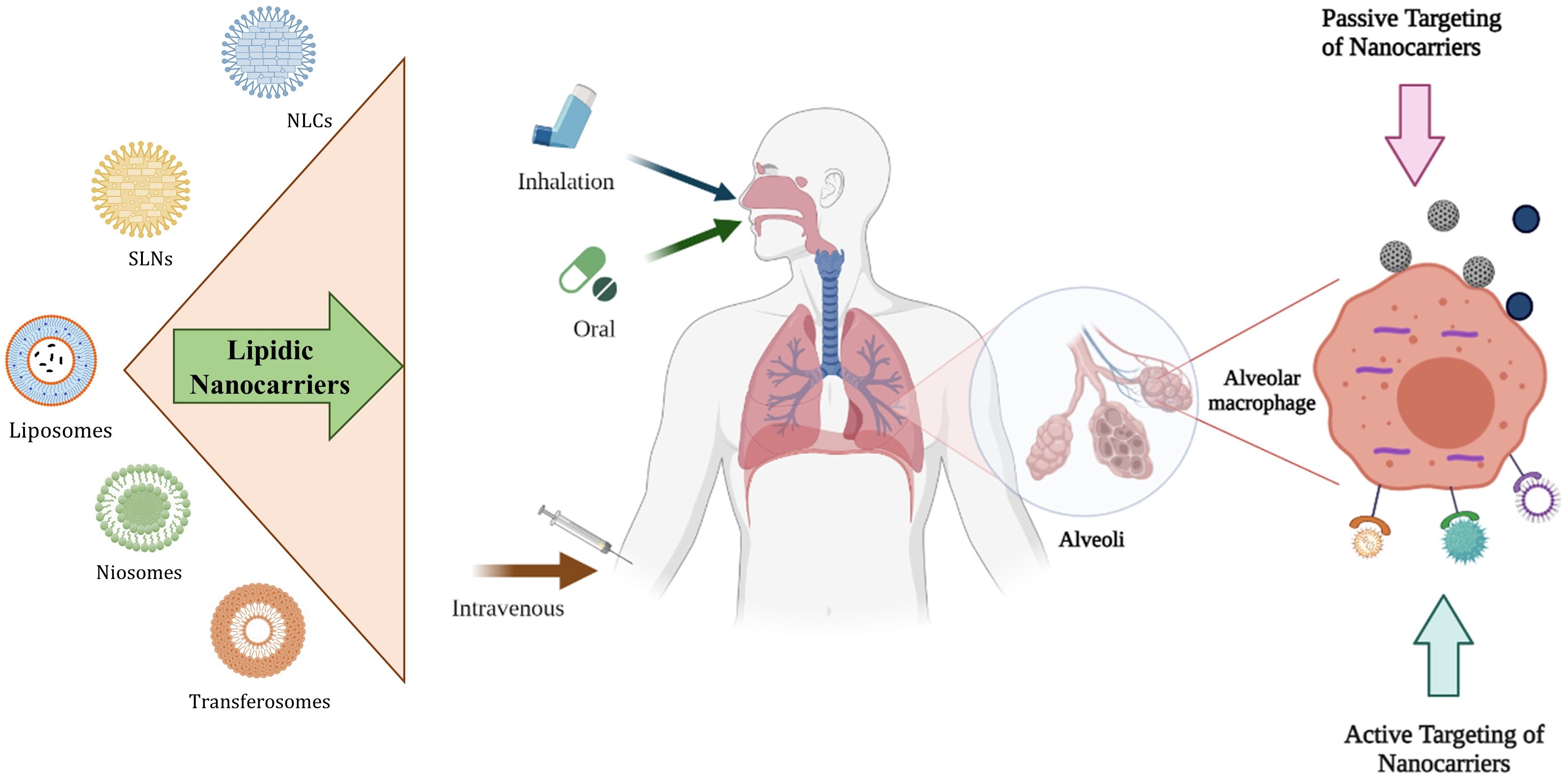

Supplement: Supplementary file 2 [file Image2.JPEG]
